# Seascape Genetics of a Globally Distributed, Highly Mobile Marine Mammal: The Short-Beaked Common Dolphin (Genus *Delphinus*)

**DOI:** 10.1371/journal.pone.0031482

**Published:** 2012-02-02

**Authors:** Ana R. Amaral, Luciano B. Beheregaray, Kerstin Bilgmann, Dmitri Boutov, Luís Freitas, Kelly M. Robertson, Marina Sequeira, Karen A. Stockin, M. Manuela Coelho, Luciana M. Möller

**Affiliations:** 1 Centro de Biologia Ambiental, Faculdade de Ciências, Universidade de Lisboa, Lisbon, Portugal; 2 Department of Biological Sciences, Macquarie University, Sydney, New South Wales, Australia; 3 School of Biological Sciences, Flinders University, Adelaide, South Australia, Australia; 4 Graduate School of the Environment, Macquarie University, Sydney, Australia; 5 Centro de Oceanografia, Faculdade de Ciências, Universidade de Lisboa, Lisbon, Portugal; 6 Museu da Baleia da Madeira, Caniçal, Madeira, Portugal; 7 National Marine Fisheries Service, Southwest Fisheries Science Center, La Jolla, California, United States of America; 8 Instituto de Conservação da Natureza e Biodiversidade, Lisbon, Portugal; 9 Coastal-Marine Research Group, Institute of Natural Sciences, Massey University, Auckland, New Zealand; Barnard College, Columbia University, United States of America

## Abstract

Identifying which factors shape the distribution of intraspecific genetic diversity is central in evolutionary and conservation biology. In the marine realm, the absence of obvious barriers to dispersal can make this task more difficult. Nevertheless, recent studies have provided valuable insights into which factors may be shaping genetic structure in the world's oceans. These studies were, however, generally conducted on marine organisms with larval dispersal. Here, using a seascape genetics approach, we show that marine productivity and sea surface temperature are correlated with genetic structure in a highly mobile, widely distributed marine mammal species, the short-beaked common dolphin. Isolation by distance also appears to influence population divergence over larger geographical scales (i.e. across different ocean basins). We suggest that the relationship between environmental variables and population structure may be caused by prey behaviour, which is believed to determine common dolphins' movement patterns and preferred associations with certain oceanographic conditions. Our study highlights the role of oceanography in shaping genetic structure of a highly mobile and widely distributed top marine predator. Thus, seascape genetic studies can potentially track the biological effects of ongoing climate-change at oceanographic interfaces and also inform marine reserve design in relation to the distribution and genetic connectivity of charismatic and ecologically important megafauna.

## Introduction

Identifying environmental conditions underlying the division of species into smaller units is central for understanding ecological and evolutionary processes and for the conservation management of biodiversity. In highly mobile species that are distributed across continuous environments with few barriers to dispersal, it is expected that persistent gene flow will stifle genetic differentiation and speciation. Nevertheless, there is growing recognition that gene flow can be limited even in the absence of geographical barriers, both in terrestrial and aquatic environments [1], [2]. A detailed knowledge of how landscape characteristics structure populations has therefore become an important focus of molecular ecological research [3], leading to the emerging field of landscape genetics [3], [4]. This multidisciplinary approach aims to complement genetic data with lines of evidence from other areas such as spatial statistics and landscape ecology in order to understand the effects of the landscape on the spatial distribution of genetic diversity [3], [5], [6]. Although extensively applied in terrestrial systems, this approach has been used less frequently in the marine environment [4]; but see [7], [8].

The study of connectivity in marine systems can be challenging due to the absence of obvious barriers to dispersal and generally large population sizes of marine organisms that often resist genetic divergence, leading to low statistical power to detect population structure [8], [9]. Therefore, the use of an integrative approach such as the one used in landscape genetics (or ‘seascape genetics’ when applied to the marine environment) has provided valuable insights into which factors may be shaping genetic structure in the world's oceans [7], [10]. Biogeographic barriers and environmental variables such as ocean currents, upwelling, variation in sea surface temperature and salinity are some of the factors that have been proposed to explain genetic diversity and structure in marine organisms [9], [10], [11]. However, most of these studies have been conducted in organisms with larval dispersal. In active marine dispersers such as sharks and dolphins, where dispersal potential is dependent upon individual vagility, the interplay of environmental features and genetic structure has remained largely untested (but see [12]). Although differences in salinity, temperature and productivity levels have been suggested to explain genetic discontinuities in dolphins [13], [14], [15], [16], a direct relationship between such oceanographic features and genetic structure has only been recently evaluated for two coastal dolphin species with limited distribution: the franciscana (*Pontoporia blainvillei*) [12] and the humpback dolphin (*Sousa chinensis*) [17]. These authors found that heterogeneity in chlorophyll concentration, water turbidity and temperature likely influenced the occurrence of genetically distinct populations of these species along the coast of Argentina and in the Western Indian Ocean, respectively.

In this study we use as model a highly mobile, widely distributed cetacean species belonging to the genus *Delphinus*, the short-beaked common dolphin. Common dolphins occur in all oceans from tropical to temperate waters. Two species and four subspecies are currently recognized: the short-beaked common dolphin, *Delphinus delphis* Linnaeus, 1758, distributed in continental shelf and pelagic waters of the Atlantic and Pacific Oceans; the long-beaked common dolphin, *Delphinus capensis* Gray, 1828, distributed in nearshore tropical and temperate waters of the Pacific and southern Atlantic waters; *D. d. ponticus* Barabash, 1935, restricted to the Black sea; and *D. c. tropicalis* van Bree, 1971, restricted to the Indian Ocean [18]. However, due to discordance between morphological and genetic characters, the phylogenetic relationships and taxonomy within the genus, particularly in regard to the specific status of the long-beaked form, are still under debate (Amaral *et al.* unpublished data; [19]).

Short-beaked common dolphins are known to occur in large groups of dozens to hundreds of individuals. Although their social structure is still poorly understood, individuals seem to group irrespective of genetic relationships, with possible gender and age segregation [20]. However, there is a gap in knowledge if these findings are representative for common dolphins in other geographic regions. The movements of common dolphins are thought to be largely determined by those of their potential prey (e.g. [21]) and their diet varies between locations and seasons [21], [22]. Nonetheless, they generally depend on small, mesopelagic shoaling fishes such as scombroids and clupeoids, and squids [21], [22]. It has been suggested that short-beaked common dolphins often prefer specific water masses [15], [23], [24] and in the Eastern Tropical Pacific they occur preferentially in upwelling-modified waters [23].

Genetic studies conducted so far have shown significant genetic differentiation among populations inhabiting different oceans and different coasts of the Atlantic Ocean [19], [25]. However, within each side of the Atlantic Ocean, no genetic structure has been detected, suggesting a lack of strong dispersal barriers in these areas [25], [26]. Within the Pacific Ocean, results from regional studies have reported fine-scale (≤1000 kms) population genetic structure in short-beaked common dolphins occurring off the USA coast (Chivers *et al.* unpublished data), off the Eastern [15] Australian Coast and around New Zealand (Stockin *et al.* unpublished data). Particular oceanographic characteristics, such as ocean currents and temperature and salinity differences have been pointed out as likely factors limiting movement of short-beaked common dolphins (Chivers *et al.* unpublished data; [15], [27]). However, a direct evaluation of the influence of oceanographic variables on the genetic structure of this species has never been carried out.

Our aim is to assess the relative influence of key oceanographic variables on population subdivision of short-beaked common dolphins at a range of medium to large spatial scales, including within ocean basins and across oceans. To achieve this aim we have sampled populations inhabiting the Atlantic, Pacific and Indian Oceans and used remote sensing data under a seascape genetics approach. The global distribution, high mobility, and putatively close association of short-beaked common dolphins with water masses, makes them an excellent model species to test for interactions between variation in environmental factors and genetic structure, contributing towards an understanding of ecological processes affecting population connectivity in the sea.

## Methods

### Ethics Statement

This study was conducted according to relevant national and international guidelines. No ethics approval was considered necessary because the animals were not handled directly. Permissions for collecting samples were obtained separately in countries where it was required (Macquarie University Animal Ethics Committee, Australia; Southwest Fisheries Science Center Ethics Advisory Committee, USA; Institute for Nature Conservation and Biodiversity, Portugal; and Department of Conservation, New Zealand). CITES permits numbers used to export/import samples were: 07US168545/9, 08US198270/9, 2009-AU-550713, 2009-AU-57-1209, 10NZ000011, PT/CR-0060/2009, PT/LE-0043/2009, PT/CR-005372009, PT/CR-0054/2009, PT/CR-0055/2009, PT/CR-0056/2009, PT/CR-0057/2009, PT/CR-0058/2009, PT/CR-0059/2009.

### Sampling

**Figure 1:**
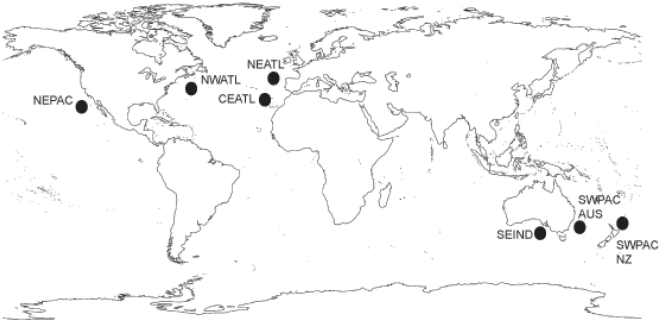
Oceanic regions sampled. Map showing sampling locations for the short-beaked common dolphin populations analysed in this study. (NEPAC – Northeast Pacific; NWATL – Northwest Atlantic; CEATL – Central eastern Atlantic; SEIND – Southeast Indian Ocean; SWPAC_AUS – Southwest Pacific Australia; SWPAC_NZ – Southwest Pacific New Zealand).

We used samples from seven oceanic regions (Figure 1): the Northeast Atlantic (NEATL), *n* = 75; the Central Eastern Atlantic (CEATL), *n* = 29; the Northwest Atlantic (NWATL), *n* = 38; the Northeast Pacific (NEPAC), *n* = 40; the Southwest Pacific, *n* = 35 (encompassing eastern Australian waters, SWPAC_AUS) and *n* = 39 (encompassing New Zealand waters, SWPAC_NZ) and the Southeast Indian Ocean (southern Australian waters, SEIND), *n* = 27 (Table 1). All tissue samples were obtained from either stranded animals (103 samples) or from skin biopsies (178 samples) collected from free-ranging dolphins. Tissues were stored either in ethanol or in 20% DMSO/saturated NaCl.

**Table 1:** Genetic diversity measures of 14 microsatellite loci for the short-beaked common dolphin populations analysed in this study.

Region	*N*	*N*a	*A*r	*H*_E_	*H*_O_	*F*_IS_
NE Atlantic (NEATL)	75	10.500	8.371	0.789	0.774	0.020
CE Atlantic (CEATL)	29	8.214	7.511	0.739	0.687	0.072
NW Atlantic (NWATL)	38	9.286	8.184	0.785	0.745	0.051
NE Pacific (NEPAC)	40	11.643	9.424	0.784	0.730	0.069*
SW Pacific Australia (SWPAC_AUS)	35	10.643	8.485	0.782	0.726	0.073*
SW Pacific New Zealand (SWPAC_NZ)	39	10.500	9.130	0.792	0.697	0.121*
SE Indian (SEIND)	25	7.571	7.163	0.700	0.696	0.006
Total/Mean	281	9.765	8.324	0.767	0.722	

*N* - sample size; *N*a - mean number of alleles; *A*r - allelic richness; *H*_E_ - expected heterozygosity; *H*_O_ - observed heterozygosity; *F*_IS_ - inbreeding coefficient.

*value statistically significant at *P*<0.05.

### DNA extraction and microsatellite genotyping

Genomic DNA was isolated from skin or muscle using a standard proteinase K digestion and two phenol-chloroform and one chlorofom-isoamyl extractions followed by ethanol precipitation [28] for samples originated from stranded animals or, alternatively, using a salting-out protocol [29] for samples originated from biopsies. DNA quality and concentration was verified using Thermo Scientifc NanoDrop 1000 Spectrophotometer (Thermo Fisher Scientific Inc.). Samples from NEPAC and NWATL were provided as DNA by the Southwest Fisheries Science Center, Marine Mammal and Turtle Research Sample Collection (SWFSC-NOAA, La Jolla, CA).

All samples were genotyped at 14 polymorphic microsatellite loci: 7 tetranucleotide (Tur4_80, Tur4_87, Tur4_92, Tur4_105, Tur4_141, Tur4_142; [30] and Dde59 [31] and 7 dinucleotide (Dde66, Dde70; [31]), KW2, KW12 [32], EV1 [33], MK6 and MK8 [34]. The forward primer for each primer pair was labelled with a M13 tag [35]. Fluorescent dyes were also labelled with the M13 tag. Amplification reactions contained 50–100 ng DNA, 1× GoTaq® reaction buffer (Promega), 2.5 mM MgCl_2_, 0.2 mM dNTPs, 0.1 µM of each primer and 1 U GoTaq® *Taq* DNA polymerase (Promega). The thermal cycler profile for the tetranucleotide loci and Dde66 and Dde70 consisted of initial denaturation at 94°C for 3 min followed by a touchdown profile for 5 cycles with the annealing temperature starting at 63°C and decreasing 2°C per cycle, followed by 30 cycles with an annealing temperature of 53°C, and a final extension step at 72°C for 10 min. The tetranucleotide loci were amplified in multiplex after optimization. For the remaining dinucleotide loci, conditions followed the original publications. All reactions included both positive and negative controls. Following amplification, samples were mixed with an internal size standard (LIZ 500) and run on an ABI 3130 Genetic Analyzer. The GeneMapper v.4.1 software (Applied Biosystems, CA) was used for sizing of allele fragments.

### Data analysis

#### Genetic diversity

The program Micro-checker v.2.2.3 [36] was used to check for the presence of genotyping errors such as scoring errors due to stuttering, large allele dropout or evidence for null alleles. Departures from Hardy-Weinberg Equilibrium were tested for each population using the Fisher exact test in Genepop v.4.0 [37]. Genepop was also used to test for linkage disequilibrium between loci. Samples were grouped into 7 putative populations according to their geographical origin as described above. Genetic diversity measures such as mean number of alleles per locus and observed (*H*_O_) and expected (*H*_E_) heterozygosities were calculated in Arlequin v.3.5.1 [38] and allelic richness (*A*_R_) calculated using FSTAT v.2.9.3 [39].

#### Genetic differentiation

Three different measures of population differentiation were used: the fixation index *F*_ST_, estimated using FSTAT [39]; the analogous *R*_ST_, estimated using Genepop v.4.0 [37]; and the statistic Jost's *D* [40], estimated using SMOGD v.1.2.5 [41]. The latter has been shown to provide a more accurate measure of differentiation when using highly polymorphic microsatellite loci [40]. Additionally, we tested for a mutation effect on genetic structure by randomly reassigning allele sizes while keeping allele identity the same [42]. The test was conducted in spagedi v.1.3 through 10,000 permutations. *R*_ST_ values significantly larger then *F*_ST_ values indicate that mutation, in addition to drift and gene flow, has contributed to frequency differences among samples, which in some cases can be interpreted as phylogeographic signal [42].

In order to visualize relationships among putative populations based on genetic variation, we performed a principal component analysis (PCA) on a table of standardised allele frequencies using the adegenet and ade4 packages in R [43]. In addition, we performed an analysis of nonmetric multidimensional scaling (MDS, [44]) on each of the genetic distance matrices using the primer computer package [45].

An analysis of molecular variance, AMOVA [46] was conducted in Arlequin to assess population structure. Different hierarchical levels were tested, considering differences occurring between populations in different oceans and within the same ocean basin.

A Bayesian approach to identify the number of populations (*K*) present in the dataset was implemented in the program STRUCTURE v.2.3.3 [47], [48]. The admixture and the correlated allele frequencies models were implemented since we expect that allele frequencies in the different populations are likely to be similar due to migration or shared ancestry. Sampling locations were used as prior to help detect population structure [49]. Ten independent runs of *K* between 1 and 8 were run with 400 000 “burn in” and 4 million MCMC replicates. The maximum log-likelihood values from all runs corresponding to each given *K* were checked for consistency and averaged. The *K* with the highest averaged maximum log-likelihood was considered the most likely number of clusters that better explains our dataset. *CLUMMP* v.1.1.2 [50] was used to summarize parameters across 10 runs and *distruct* v.1.1 [51] was used to produce the corresponding graphical output.

#### Isolation by distance

Isolation by distance (IBD) was evaluated using a Mantel test implemented in the program IBDWS v.3.16 [52]. Genetic distance matrices given by *F*_ST_/(1−*F*_ST_) were regressed against the logarithm of geographical distances following a two-dimensional model [53]. *R*_ST_ and Jost's *D* values were also used. Geographic distances were measured in Google Earth by using set points and measuring either straight-line distance across oceans, or the shortest geographical distance along continental margins. The set points were chosen so as to represent the middle point of the area of distribution where the samples were collected.

#### Environmental predictors of genetic structure

Three different oceanographic variables were used as predictors of the observed genetic differences between short-beaked common dolphin populations. These were night-time sea surface temperature (SST, °C), chlorophyll concentration (CHL, mg/m^3^) and water turbidity measured as diffuse attenuation coefficient at 490 nm (KD490, m^−1^). These variables, here obtained from remote sensing data, have been previously related to habitat heterogeneity [54] and associated with genetic differences in other dolphin species [17]. Furthermore, the oceanographic variables chosen have a wide geographic coverage through remote sensing, making them ideal for a global approach. Seven oceanic regions, corresponding to the sampling areas for short-beaked common dolphins, were used for the extraction of these oceanographic variables to assess association with patterns of genetic differentiation. Polygons were defined considering the possible range of common dolphins within that oceanic region, with the last side being the coastline. For NWATL the area was defined between 46°N, 38°N and 57°W; for CEATL between 34°N, 32°N and 16°W; for NEATL between 60°N, 35°N and 0°; for NEPAC between 45°N, 25°N and 108°W; for SWPAC_NZ between 32°S, 44°S and 180°W; for SWPAC_AUS between 26°S, 44°S and 156°E; and for SEIND between 31°S, 37°S and 140°E. In order to account for possible influence of area choice in the final results, areas restricted to where samples from free-ranging animals originally came from or from published distributional data were considered and re-analysed. Since no differences were found in the final results, only analyses including the areas defined above are presented, which account for a possible wider ranging distribution of common dolphins. Monthly averaged data of the three variables, with a 4 km spatial resolution was obtained from Ocean Color Web (http://oceancolor.gsfc.nasa.gov/) for the period from July 2002 to October 2010 and processed using MATLAB software (www.mathworks.com). Data collected during this time period provide a characterization of the oceanographic features for each region and are robust to inter-annual oscillations (Supplementary Material, Figure S1). Data analysis included the construction of temperature, chlorophyll and turbidity maps for each region, where each pixel of the map corresponds to the eight-year average value for a 4 km grid. These maps were visually inspected to detect geographical areas of environmental heterogeneity. Monthly averages for each oceanic region were then statistically analysed using a paired t-test to detect differences among those regions. Total averages for the 8 year-period for each factor and each sampled region were subsequently used to examine environmental and genetic associations (details below). Environmental distances were calculated as pairwise differences in mean temperature, chlorophyll and turbidity between regions. Pairwise *F*_ST_, *R*_ST_ and Jost's *D* were used as genetic distances.

All analyses were carried out at different spatial scales: at a large scale, all oceans included; each ocean considered in separate, i.e. all populations within the Atlantic and all populations within the Pacific Ocean and the population in the Southeast Indian Ocean; and at a medium scale, the North and Central Atlantic populations (hereinafter referred to as North Atlantic) and the South Pacific and Southeast Indian Ocean populations (hereinafter referred to as South Indo-Pacific).

#### Seascape genetics

Associations between genetic and environmental factors were examined using a hierarchical Bayesian method implemented in GESTE [55], which estimates individual *F*_ST_ values for each local population and then relates them to environmental factors via a generalized linear model. Here we used 10 pilot runs of 1,000 iterations to obtain the parameters of the proposal distribution used by the MCMC, and an additional burn-in of 5×10^6^ iterations with a thinning interval of 20. The model with the highest posterior probability is the one that best explains the data [55].

Additionally, we used the BIOENV procedure of [56] as implemented in primer v.5 [45] and as described in [57] to examine which predictor variable would provide the best model to explain the population genetic structure observed in the data. This procedure calculates the value of Spearman's rank correlation coefficient (ρ) between a genetic distance matrix (response matrix) with a distance matrix calculated as the Euclidean distance among one or more predictor variables. It then calculates the value of ρ using every possible combination of predictor variables until it finds the “best fit”, corresponding to the combination of predictor variables whose Euclidean distance matrix yields the highest value of ρ [56]. We used three different response matrices corresponding to *F*_ST_, *R*_ST_ and Jost's *D* distance matrices to identify the best one, two or three-variable fits.

Mantel tests [58] were also used to test for correlations between the pairwise genetic and environmental distances. Partial Mantel tests were used to control the effect of geographical distances in these potential correlations. These tests were performed using the package vegan in R.

## Results

### Genetic Diversity

In total 281 short-beaked common dolphin samples were genotyped at 14 microsatellite loci (Table 1). Results from Micro-Checker and the Fisher exact test suggested deviations from Hardy-Weinberg equilibrium (HWE) in 4 loci. Two of these (Tur91 and Tur80) showed deviations in only one population each and were therefore included in subsequent analyses, whereas the other two (Tur141 and Dde66) showed deviations in 4 and 2 populations, respectively. These deviations are due to a deficit of heterozygotes (significant *F*_IS_ values, Table 1). To test whether results would be affected by the inclusion of these two loci, estimates of genetic variability and differentiation were carried out with and without them. Since no major differences in results were observed (data not shown), all 14 loci were used in subsequent analyses. These deviations are likely not related with the fact that some samples originated from strandings and others from biopsies. In fact, it has been recently shown that no apparent differences occur when testing population structure in common dolphins using samples originated from carcasses or from free-ranging dolphins [59].

Levels of genetic diversity, given by mean number of alleles, allelic richness and expected and observed heterozygosities were high for most populations (Table 1). Significant *F*_IS_ values were obtained for populations from NE Pacific and SW Pacific Australia and New Zealand, which can be due to the presence of population sub-structure (i.e. Wahlund effect). In fact, this is known to be the case for common dolphins inhabiting those regions ([15], [27]; Stockin *et al.* unpublished).

### Genetic differentiation

Pairwise *F*_ST_ and *R*_ST_ comparisons showed significant levels of differentiation among all putative populations (Table 2), although the extent of that differentiation differed for each index. Jost's *D* values tended to be higher than *F*_ST_ and *R*_ST_ values. *R*_ST_ also tended to be higher than *F*_ST_. Since *R*_ST_ is based on allele size, the differences observed indicate that mutation, in addition to drift or gene flow may be affecting the differentiation between these populations. This result was confirmed using spagedi. The overall *R*_ST_ value was significantly higher than the overall *F*_ST_ value (*P* = 0.042).

**Table 2:** Pairwise fixation index values obtained between short-beaked common dolphins populations for 14 microsatellite loci.

a) *F*_ST_						
	NEATL	CEATL	NWATL	NEPAC	SWPACAUS	SWPACNZ
NEATL						
CEATL	0.0150*					
NWATL	0.0051*	0.0151*				
NEPAC	0.0313*	0.0439*	0.0284*			
SWPACAUS	0.0267*	0.0464*	0.0228*	0.0117*		
SWPACNZ	0.0268*	0.0471*	0.0239*	0.0211*	0.0137*	
SEIND	0.0680*	0.0896*	0.0716*	0.0663*	0.0473*	0.0386*

b) *R*_ST_						
	NEATL	CEATL	NWATL	NEPAC	SWPACAUS	SWPACNZ
NEATL						
CEATL	0.0099*					
NWATL	−0.0026	0.0069*				
NEPAC	0.0341*	0.0434*	0.0335*			
SWPACAUS	0.0122*	0.0280*	0.0059*	0.0114*		
SWPACNZ	0.0373*	0.0671*	0.0336*	0.0720*	0.0668*	
SEIND	0.0430*	0.0656*	0.0419*	0.0976*	0.0497*	0.0923*

c) Jost's *D*						
	NEATL	CEATL	NWATL	NEPAC	SWPACAUS	SWPACNZ
NEATL						
CEATL	0.0082					
NWATL	0.0119	0.0103				
NEPAC	0.1136	0.1422	0.1090			
SWPACAUS	0.0687	0.1142	0.0673	0.0293		
SWPACNZ	0.0921	0.1398	0.0814	0.0234	0.0135	
SEIND	0.1479	0.1795	0.1670	0.1542	0.0835	0.0736

a) *F*_ST_; b) *R*_ST_ and c) Jost's *D*.

Taken as a whole, the fixation indices showed high levels of differentiation between short-beaked populations inhabiting different ocean basins. The SEIND and NEPAC populations showed the highest levels of differentiation when compared with all other short-beaked populations. Contrasting to the inter-ocean basin differentiation, lower levels of differentiation were observed between short-beaked populations inhabiting the same ocean basins.

**Figure 2:**
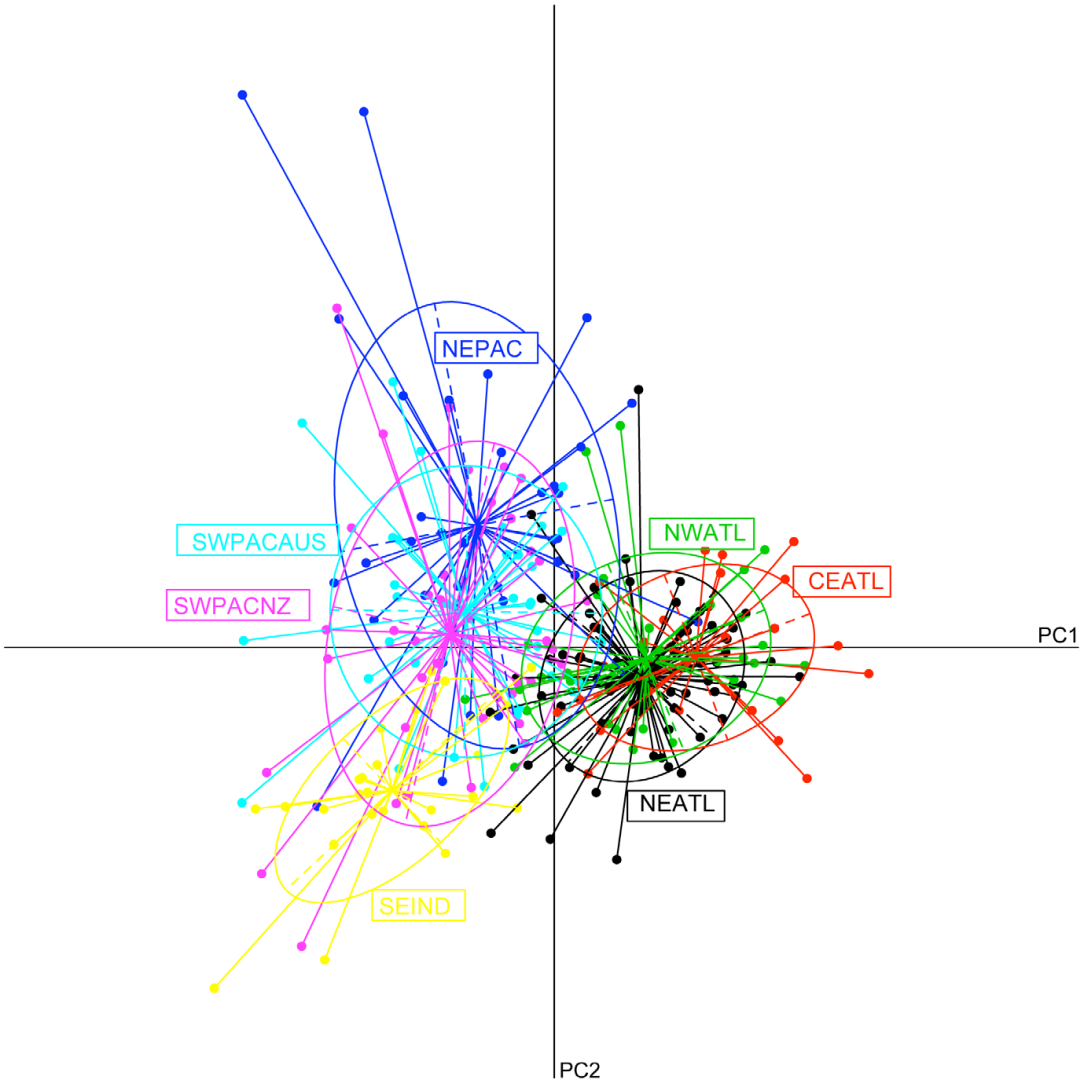
Principal component analysis. Principal component analysis (PCA) performed on a table of standardised allele frequencies based on 14 microsatellite loci of the short-beaked populations analysed in this study.

The first two principal components of the PCA analysis explained 84.35% of the variance in allele frequencies among putative populations (Figure 2). The first principal component shows a clear separation between populations inhabiting the Indo-Pacific and the Atlantic Oceans. The second principal component further shows some structure within the Indo-Pacific region, with the SEIND and NEPAC populations appearing separated from the SWPAC_AUS and SWPAC_NZ populations.

**Figure 3:**
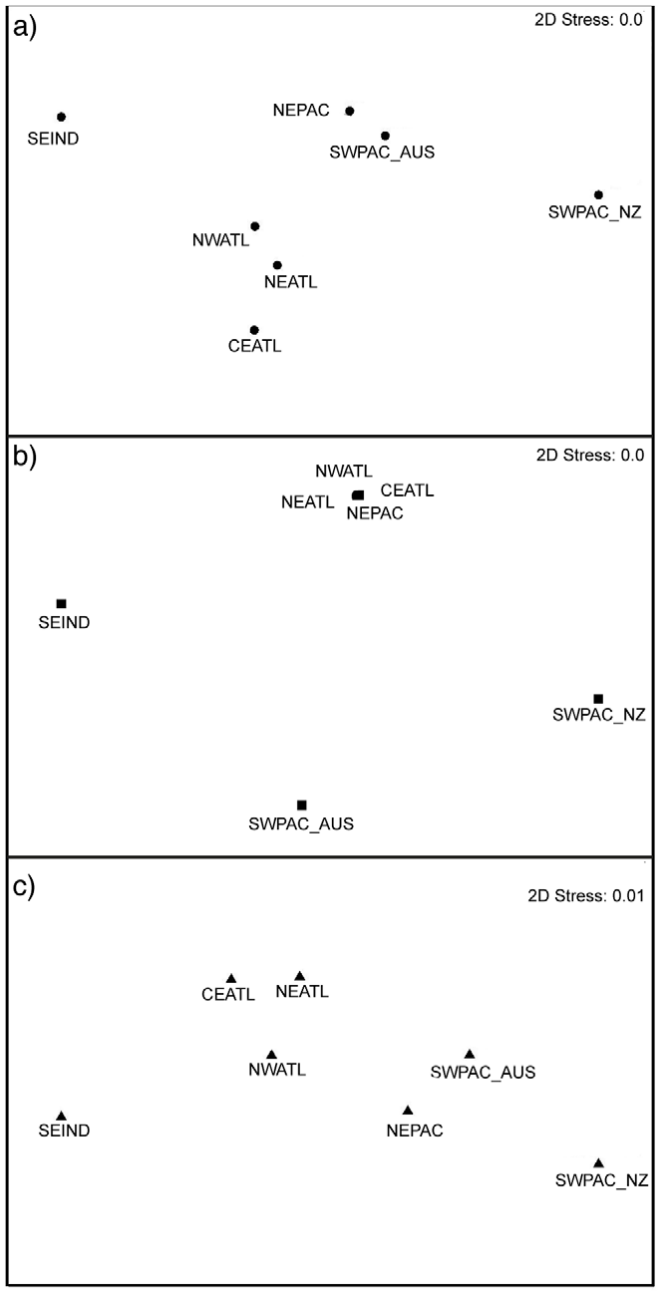
Non-metric MDS. Non-metric MDS plots of short-beaked common dolphin populations on the basis of genetic distances using a) *F*_ST_, b) *R*_ST_ or c) Jost's *D*. Stress values are indicated.

Non metric MDS analyses using the three different genetic indices also show a clear separation from populations inhabiting the Atlantic, the Pacific and Indian oceans, with the exception of the analysis using *R*_ST_, which grouped the NEPAC population with Atlantic ones (Figure 3). The analyses using *F*_ST_ and Jost's *D* show a closer proximity among the short-beaked populations inhabiting the North Atlantic, and also of the populations inhabiting the Pacific Ocean.

**Figure 4:**
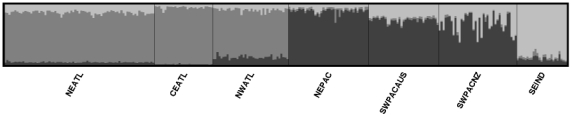
Number of clusters found for short-beaked common dolphin populations. Results from the program STRUCTURE showing individual assignment values for *K* = 3. Each colour depicts the relative contribution of each of the three clusters to the genetic constitution of each individual.

Results obtained in STRUCTURE using the correlated allele frequency model resulted in a peak of maximum ln *P*(*K*) at *K* = 3 (Figure 4, Supplementary Table S2). These clusters correspond to populations inhabiting the three ocean basins: the Atlantic (including the NEATL, NWATL and CEATL populations), the Pacific (including the NEPAC, SWPAC_AUS and SWPAC_NZ populations) and the Indian Ocean including the SEIND population (Figure 4).

The AMOVA analysis showed that the highest levels of differentiation were obtained when populations were divided by eastern versus western regions within ocean basins (*F*_CT_ = 0.03425, *P*<0.0001) (Table 3).

**Table 3:** Analysis of hierarchical variance (AMOVA) results obtained for the short-beaked common dolphin populations.

Source of variation	%variation	*F*-statistics	*P*
Among ocean basins	2.71	*F*_CT_ = 0.02710	0.0000
Among groups within populations	1.35	*F*_SC_ = 0.01386	0.0000
Within populations	95.94	*F*_ST_ = 0.04058	0.0000
Among regions	1.92	*F*_CT_ = 0.03425	0.0001
Among groups within populations	1.5	*F*_SC_ = 0.01532	0.0000
Within populations	96.58	*F*_ST_ = 0.03425	0.0000

### Isolation by distance

The relationship between geographic and genetic distance was only observed when populations inhabiting all oceans were considered in the analysis and when *F*_ST_ and Jost's *D* values were used (Table 4). This relationship was not detected when *R*_ST_ values were used, nor when finer spatial scales were considered.

**Table 4:** Summary results for Isolation by Distance tests conducted for all short-beaked common dolphin populations in all oceans, for North Atlantic populations only, for Pacific populations only, and for South Indo-Pacific populations only.

	*P*	*r* (slope)	*R* ^2^
All oceans			
Fst	**0.0196**	0.0502	0.1560
Rst	0.9072	−0.0657	0.0416
Jost's *D*	**0.0091**	0.1240	0.4660
North Atlantic			
Fst	0.4995	−0.0211	0.2010
Rst	0.8351	−0.0239	0.4210
Jost's *D*	0.3316	0.0068	0.7740
Pacific			
Fst	0.3364	0.0573	0.0483
Rst	0.6241	−0.0840	0.0024
Jost's *D*	0.3328	0.1410	0.1150
South Indo-Pacific			
Fst	0.3310	0.0984	0.7860
Rst	0.4980	0.1209	0.1130
Jost's *D*	0.3321	0.2137	0.8760

Values in bold were statistically significant (*P*<0.05).

### Oceanographic predictors

**Figure 5:**
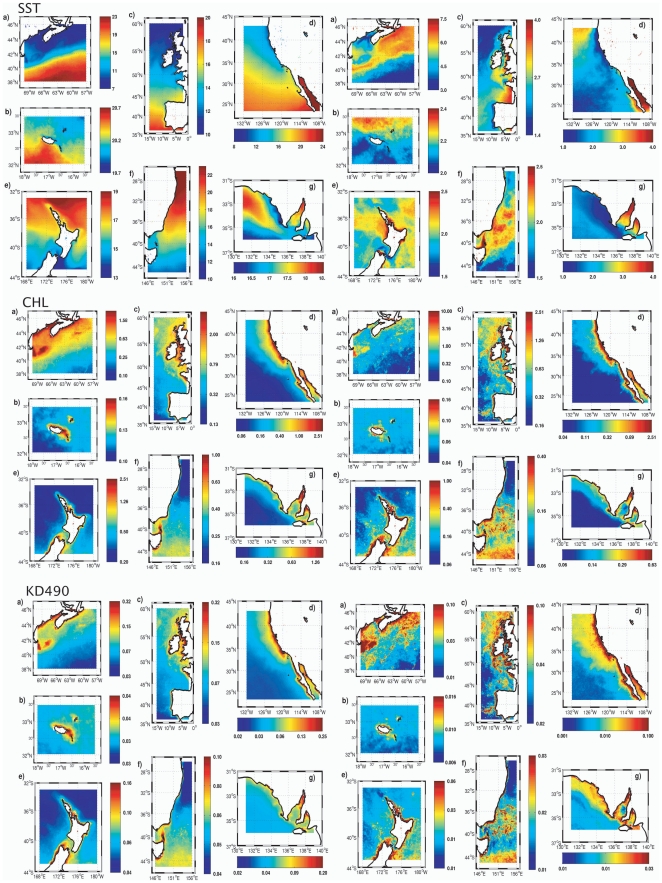
Oceanographic predictors for each oceanic region. Regional maps showing 8-year average values for sea surface temperature (SST), chlorophyll concentration (CHL) and water turbidity (KD490) on the left and standard deviation values on the right for the oceanic regions where the short-beaked common dolphin populations analysed in this study were sampled: a) Northwest Atlantic; b) Central eastern Atlantic; c) Northeast Atlantic; d) Northeast Pacific; e) Southwest Pacific New Zealand; f) Southwest Pacific Australia; g) Southeast Indian.

Data on sea surface temperature (SST), chlorophyll concentration (CHL) and water turbidity (KD490) was gathered for the seven oceanic regions where short-beaked common dolphins were sampled: NEATL, CEATL, NWATL, NEPAC, SWPAC_AUS, SWPAC_NZ and SEIND (Figure 5). Paired t-tests showed significant differences in the 8 year average values of SST between most regions with exception of the comparison between NEATL and NWATL, between NEPAC and SWPAC (both AUS and NZ), and between NEPAC and SEIND, where differences were not statistically significant (*P*<0.01, see Supplementary Material, Table S1). In the SST maps, all regions are heterogeneous, having regions of colder and warmer waters (Figure 5). Nevertheless, NEATL and NWATL regions are dominated by colder waters when compared with other regions, which are dominated by warmer waters, such as SWPAC_AUS and SWPAC_NZ. Significant differences were not detected in mean CHL values between NEPAC and SWPAC (both AUS and NZ) and between NEPAC and SEIND, as well as among SEIND, SWPAC_AUS and SWPAC_NZ. All other comparisons were significant. Despite this, in the CHL maps, clear differences can be seen among the regions located in the Pacific Ocean. Chlorophyll concentrations are higher in the NEPAC region closer to the coast when compared to the SWPAC_AUS and SWPAC_NZ regions. Regarding turbidity mean values, these were only not significant in the comparisons among SWPAC_AUS, SWPAC_NZ and SEIND (Table S1). Patterns seen in the maps are similar to the ones obtained for the CHL maps (Figure 5).

### Seascape genetics

Hierarchical Bayesian analyses implemented in GESTE identified the model including the constant as the best one in all spatial scales considered (Table 5). The second best model for all analyses was the one including KD490, though the third and fourth models (including CHL and SST) all had very similar posterior probability values. Higher posterior probabilities were obtained when medium spatial scales were analysed. Positive signals of the regression coefficients were obtained for the association between CHL and genetic differentiation in the Pacific Ocean and South Indo-Pacific Ocean populations, and for the association between KD490 and genetic differentiation in the Pacific Ocean populations (Table 5). Regarding SST, positive signals of the regression coefficients were obtained for all populations across all oceans, for the North Atlantic populations, and for the South Indo-Pacific populations (Table 5). Therefore, genetic isolation of populations within the Pacific Ocean increases with differences in CHL and KD490 among regions, whereas genetic isolation of populations within the Atlantic Ocean increases with differences in SST among regions. In the South Indo-Pacific region, both CHL and SST increase genetic isolation among populations. The percentage of variation that remained to be explained (indicated by sigma values) was however moderate (Table 5).

**Table 5:** Posterior probabilities of the four most probable models for the GESTE analysis of environmental associations with genetic structure (population specific *F*_ST_) of short-beaked common dolphins.

Model	Factors included	*P*	Coefficient	Mean	Mode	95% HPDI
All Oceans						
1	Constant	0.702	α_0_	−3.02	−3.01	−3.60; −2.43
			σ	0.591	0.378	0.125; 1.319
2	Constant, SST	0.067	α_0_	−3.01	−2.99	−3.61; −2.33
			α_1_	0.13	0.12	−0.52; 0.73
			σ	0.708	0.422	0.125; 1.70
3	Constant, CHL	0.0649	α_0_	−3	−3	−3.66; −2.36
			α_2_	−0.13	−0.11	−0.69; 0.56
			σ	0.679	0.367	0.123; 1.501
5	Constant, KD490	0.0707	α_0_	−3.03	−3.05	−3.60; −2.32
			a3	−0.1	−0.1	−0.80; 0.53
			σ	0.694	0.4	0.113; 1.726
Pacific						
1	Constant	0.628	α_0_	−3.08	−3.12	−4.02; −1.97
			σ	1.094	0.701	0.173; 2.88
2	Constant, SST	0.092	α_0_	−3.1	−3.16	−4.30; 2.02
			α_1_	−0.04	−0.12	−1.26; −1.10
			σ	1.42	0.695	0.198; 4.102
3	Constant, CHL	0.0991	α_0_	−3.04	−3.1	−4.16; −1.61
			α_2_	0.13	0.06	−1.07; 1.25
			σ	1.63	0.713	0.140; 4.47
5	Constant, KD490	0.104	α_0_	−3.04	−3.17	−4.16; −1.85
			α_3_	0.14	0.16	−1.10; 1.23
			σ	1.534	0.68	0.199; 4.601
North Atlantic						
1	Constant	0.496	α_0_	−3.25	−3.33	−4.52; −2.05
			σ	1.14	0.677	0.097; 3.27
2	Constant, SST	0.101	α_0_	−3.22	−3.28	−4.59; −1.61
			α_1_	0.29	0.31	−0.97; 1.9
			σ	1.557	0.774	0.114; 4.876
3	Constant, CHL	0.1	α_0_	−3.22	−3.3	−4.46; 1.63
			α_2_	−0.25	−0.25	−1.55; −1.08
			σ	1.547	0.783	0.135; 5.112
5	Constant, KD490	0.103	α_0_	−3.19	−3.32	−4.45; −1.65
			α_3_	−0.27	−0.29	−1.85; −1.11
			σ	1.694	0.86	0.134; 5.4
South Indo-Pacific						
1	Constant	0.501	α_0_	−2.95	−3	−4.26; −1.63
			σ	1.481	0.825	0.146; 4.305
2	Constant, SST	0.0946	α_0_	−2.87	−3.1	−4.25; 0.95
			α_1_	0.14	0.19	−1.52; 1.64
			σ	2.246	1.195	0.163; 7-064
3	Constant, CHL	0.0969	α_0_	−2.93	−2.99	−4.43; −1.06
			α_2_	0.08	0.13	−1.70; 1.65
			σ	2.331	0.933	0.169; 7.64
5	Constant, KD490	0.171	α_0_	−2.96	−3.07	−4.27; −1.61
			α_3_	−0.54	−0.59	−1.84; 0.91
			σ	1.678	0.765	0.124; 5.344

SST – sea surface temperature; CHL – chlorophyll concentration; KD490 – sea water turbidity measured as diffuse attenuation coefficient at 490 nm; α – regression coefficient; σ – estimate of the variation that remains unexplained by the regression model; HPDI – highest probability density interval.

The BIOENV procedure found strong positive correlations between oceanographic predictors and genetic differentiation for the analyses conducted at medium spatial scales (Table 6). For the populations within the Atlantic Ocean and within the South Indo-Pacific, CHL and KD490 showed stronger correlation with genetic distance. For the larger spatial scales considered (across all oceans and within the Pacific Ocean), a strong negative correlation between CHL and KD490 with rank genetic distance was found (Table 6).

**Table 6:** Results of the BIOENV procedure, showing the best fit obtained, for all short-beaked common dolphin populations, North Atlantic populations only, Pacific populations only, and South Indo-Pacific populations only, in the case of one, two and three predictor variables for each genetic distance matrix.

Number	Spearman's	Variables	Number	Spearman's	Variables
variables	rho	chosen	variables	rho	chosen
All Oceans			North Atlantic		
Fst			Fst		
1	−0.341	CHL	1	1	KD490
2	−0.356	CHL, KD490	2	1	CHL, KD490
3	−0.227	SST, CHL, KD490	3	0.5	SST, CHL, KD490
Jost's *D*			Jost's *D*		
1	−0.366	CHL	1	−0.5	KD490
2	−0.374	CHL, KD490	2	−0.5	CHL, KD490
3	−0.31	SST, CHL, KD490	3	−1	SST, CHL, KD490
Rst			Rst		
1	−0.713	CHL	1	1	SST
2	−0.703	CHL, KD490	2	1	SST, CHL
3	−0.573	SST, CHL, KD490	3	1	SST, CHL, KD490
Pacific			South Indo-Pacific		
Fst			Fst		
1	−0.314	CHL	1	1	KD490
2	−0.371	CHL, KD490	2	−0.5	CHL, KD490
3	−0.029	SST, CHL, KD490	3	−0.5	SST, CHL, KD490
Jost's *D*			Jost's *D*		
1	−0.314	CHL	1	1	KD490
2	−0.714	CHL, KD490	2	0.5	CHL, KD490
3	−0.714	SST, CHL, KD490	3	−1	SST, CHL, KD490
Rst			Rst		
1	0.029	CHL	1	0.5	KD490
2	0.086	CHL, KD490	2	0.5	SST, KD490
3	−0.2	SST, CHL, KD490	3	0.5	SST, CHL, KD490

SST – sea surface temperature; CHL – chlorophyll concentration; KD490 – sea water turbidity measured as diffuse attenuation coefficient at 490 nm.

Mantel tests and Partial Mantel tests between genetic and environmental distances were not statistically significant for any comparison, even considering different spatial scales (results not shown). Failures of these tests to detect relationships between genetic and environmental data have been previously described [60], [61] and could explain the unsuccessful use with our datasets.

## Discussion

We used a seascape approach to investigate the interaction between a set of oceanographic variables and population structure in a highly mobile, widely distributed top marine predator, the short-beaked common dolphin. We show that sea surface temperature, chlorophyll concentration and water turbidity seem to be important factors in explaining the observed patterns of genetic structure in these dolphins, more than geographical distance alone, particularly when medium spatial scales were considered.

### Genetic structure

The overall global pattern of genetic structure obtained here supports previous studies [19]: higher levels of differentiation were obtained across large geographical scales, between different ocean basins, and lower levels were obtained when medium geographical scales were considered, within the same ocean basin. While results from STRUCTURE showed a clear differentiation between ocean basins, the AMOVA analysis resulted in higher *F*_CT_ estimates for partitioning of short-beaked populations among regions within each ocean basin. The low levels of divergence found between populations inhabiting the same ocean basin may have affected the power of the program STRUCTURE to detect such differentiation, even using recently developed algorithms that account for weak differentiation [49]. Nonetheless, the PCA and the NMDS plots also indicate some level of differentiation within ocean basins, which seems to be stronger among the Pacific Ocean populations. Multivariate analysis does not require strong assumptions about the underlying genetic model, such as Hardy-Weinberg equilibrium or the absence of linkage disequilibrium [43]. The high levels of differentiation found for the SEIND population (southern Australia) were surprising given the comparatively shorter distance separating this population from the Southwest Pacific populations (off New South Wales, southeastern Australia), even considering that the region where the SEIND population was sampled (off South Australia) falls into a different biogeographic region (see [62] to the one of the SWPAC_AUS population. Such high differentiation was also reported by [27] when comparing individuals from this region to individuals from southeastern Tasmania (Southwest Pacific) – in that case oceanographic features affecting the distribution of target prey were suggested to be the likely explanation for the genetic differentiation found. Our study corroborates this previous finding (see below).

### Isolation by distance

A pattern of isolation by distance was only observed when large spatial scales were considered, indicating that the stronger genetic differentiation observed in short-beaked common dolphins from different oceans may be an effect of geographic distance. Isolation by distance has been reported for other cetacean species, such as in the harbour porpoise [63] and in bottlenose dolphins [64]. Conversely, when medium geographic scales were considered (i.e. within each ocean basin), no isolation by distance effect was detected, and genetic differentiation could be explained by oceanographic variables. This pattern has also been described for common dolphins at small geographical scales, along the eastern Australian coast [15], for bottlenose dolphins in South Australia where a temperature and salinity front coincides with the boundary between two distinct genetic populations [13], and for pilot whales, where ecological factors, such as SST, were more important in explaining genetic structure than geographic separation [14]. In franciscana and humpback dolphins, environmental factors were also more important in explaining genetic structure than distance at small geographical scales [12], [17].

### Oceanographic predictors

All oceanographic variables tested, CHL, KD490 and SST, showed an association with population genetic structure in short-beaked common dolphins. These associations were strongest at the medium spatial scales considered. In the Pacific Ocean, CHL and KD490 were the environmental predictors that were most strongly associated with increased genetic isolation in short-beaked common dolphins. Conversely, in the Atlantic Ocean, SST was the strongest predictor associated with population divergence. Although no significant statistical differences in the 8-year average values of CHL and KD490 were detected among regions in the Pacific Ocean, a visual inspection of the regional maps shows heterogeneity in these variables among regions (Figure 5). Heterogeneity in SST, CHL and KD490 is also seen among Atlantic Ocean regions, although our results suggest that only SST seems to explain genetic differentiation of short-beaked common dolphins in this area. Marine productivity and SST are important variables for habitat occupancy and dispersal in cetaceans [65], [66] and have been shown to influence population structure in Franciscana [12] and in humpback dolphins [17]. Here, we suggest that they are also important drivers of population structure in common dolphins. A direct causality is however difficult to establish. For example, it has been suggested that ecological factors such as prey behaviour rather than inherent sensitivity to environmental factors, could account for the relationship between SST and population structure in pilot whales [14], [66], [67]. Similarly, differences in prey distribution and abundance between regions rather than SST differences themselves are suggested to account for genetic differentiation of bottlenose dolphins in South Australia [13] and short-beaked common dolphins in southern [27] and southeastern Australia [15]. We suggest that a similar process may account for the patterns obtained in this study. Since dolphins feed high in the food chain, a statistical association with oceanographic variables that do not directly affect the individuals, but rather affect their prey, is expected to be weak [23]. This could also explain the fact that analyses performed in GESTE did not result in a single best-chosen model and that the percentage of variability that remained to be explained in the data was moderate.

Chlorophyll concentration, water turbidity and SST are routinely used to map ocean primary productivity (e.g. [68]). Due to the bottom-up processes that control marine ecosystems [69], these variables have been related to prey distribution and abundance, and to the occurrence of top marine predators (e.g. [70], [71]). Distribution and abundance of prey has been suggested as the main factor dictating seasonal migrations in several species of delphinids, including short-beaked common dolphin (e.g. [21]). Moreover, short-beaked common dolphins feed primarily on small mesopelagic schooling fish such as sardines and anchovies [21], [22]. These fishes are filter feeders and occur in association with nutrient rich waters (e.g. [72]), and could explain the dolphins' preference for certain oceanographic conditions.

We further suggest that a behavioural mechanism such as specialization for local resources could also explain the patterns observed. Resource specialization is a common mechanism driving population structure in delphinds [73]. Moreover, dietary segregation is known to occur in short-beaked common dolphins. In the Bay of Biscay, Northeast Atlantic Ocean, common dolphins inhabiting neritic and oceanic waters feed on different prey species [74]. Feeding specialization leading to local adaptation has also been suggested as driving speciation of the short and long-beak forms [19] and as important triggers for the process of population divergence and speciation in the genera *Tursiops* and *Stenella* [75], [76]. Perhaps the best studied example within delphinids are killer whales (*Orcinus orca*), where resource partitioning and foraging specializations of sympatric populations occurring in the North Pacific have lead to the evolution of distinct lineages [77]. Short-beaked common dolphins could therefore be locally adapted to the existent prey species and only move within certain regions following prey migration. Seasonal migrations are known to occur in the Northeast Pacific [78] and Southwest Indian Ocean [79]. Further investigation is however required to support this hypothesis.

There are also other factors that may account for population divergence in common dolphins that were not assessed in this study. Fine-scale oceanic processes, for example, have recently been suggested to affect connectivity in common dolphins [15]. A proper assessment of its direct relationship with genetic structure requires knowledge on hydrodynamic modelling and will certainly be the aim of forthcoming studies. Demographic and historical processes can also contribute to population structure and should also be integrated in future analyses.

### Implications for conservation and management

The results presented here are of particular importance for marine conservation management and design of marine protected areas (MPA). MPAs are usually designed to protect coastal regions that are either important habitats, as part of the marine ecosystem, or biodiversity hotspots [80]. Marine predators are often used as indicators for MPA design, because their protection aids in protecting the more complex environments they use [81], [82], [83]. Although several studies have described the distribution and occurrence of cetacean species in relation to different habitat variables (e.g. [84], [85], [86]), only a few have found a direct correlation between oceanographic variables and population structure [12], [17]. In this study, by showing how marine productivity correlate with population structure in short-beaked common dolphins, we highlight the importance of using seascape genetic studies to inform MPA design in relation to distribution and genetic connectivity of charismatic and ecologically important megafauna. Furthermore, we highlight how such an approach can track the biological effects of ongoing climate-change and prevent the loss of top marine predators [87].

## Supporting Information

Figure S1 **Annual fluctuation of oceanographic predictor values.** Annual average values for (a) sea surface temperature, (b) chlorophyll concentration and (c) water turbidity for the different oceanographic regions.
(PDF)

Table S1 Mean pairwise difference between average values of a) sea surface temperature (SST), b) chlorophyll concentration (CHL) and c) water turbidity (KD490) obtained for each oceanographic region where short-beaked common dolphins were sampled for this study, with significant values of paired t-tests indicated in bold.
(XLS)

Table S2 Individual runs for the Bayesian analysis implemented in the program STRUCTURE with a burn-in phase of 4×10^5^ and 4×10^6^ MCMC replicates. The log-likelihood of the data (LnP(D)) for each run and an average across 10 runs for each K are shown. The K with the highest averaged maximum log-likelihood was considered the most likely number of clusters that better explains our dataset (in bold).
(XLS)
