# Supplementary figures and images for: Seascape Genetics of a Globally Distributed, Highly Mobile Marine Mammal: The Short-Beaked Common Dolphin (Genus Delphinus)
Source: PLoS One. 2012 Feb 2;7(2):e31482. doi: 10.1371/journal.pone.0031482 (PMC3271111; doi:10.1371/journal.pone.0031482)

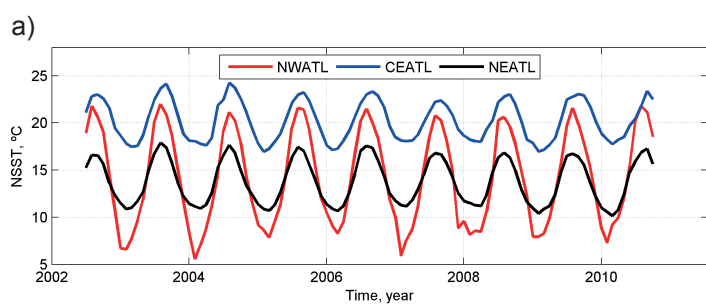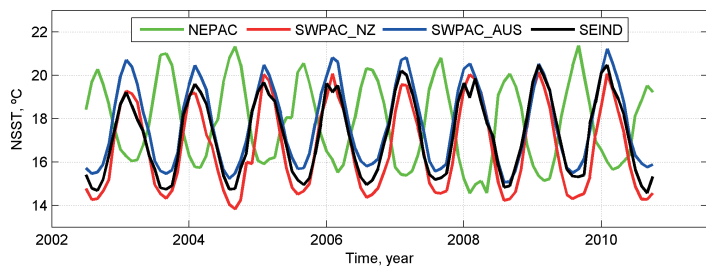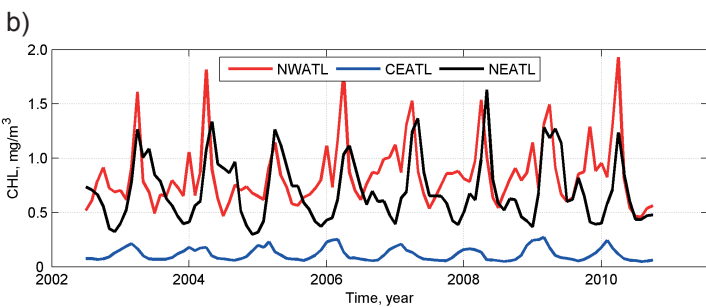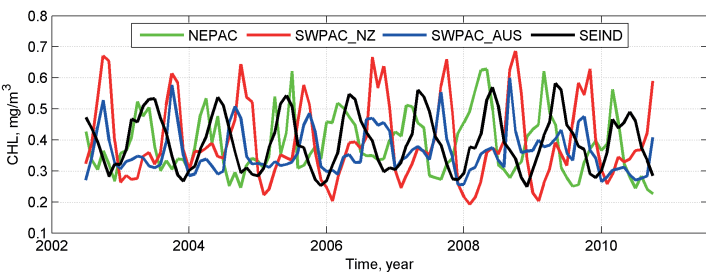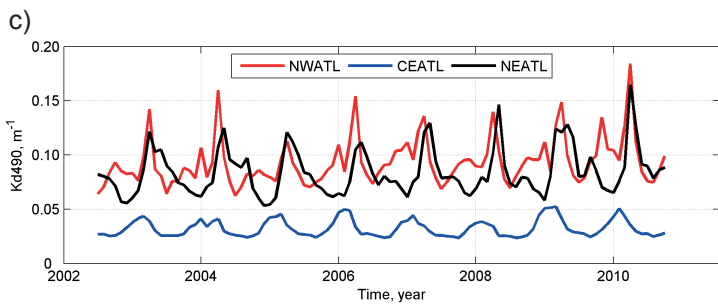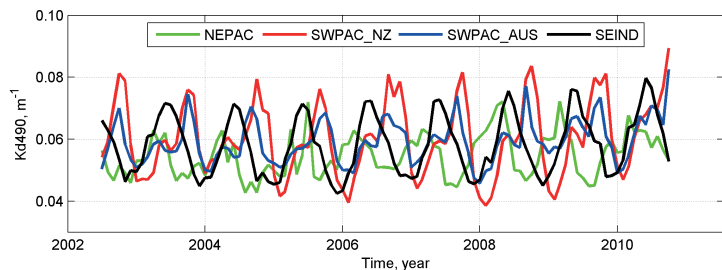

Supplement: Figure S1 — Annual fluctuation of oceanographic predictor values. Annual average values for (a) sea surface temperature, (b) chlorophyll concentration and (c) water turbidity for the different oceanographic regions. (PDF) [file pone.0031482.s001.pdf]
